# Antioxidative properties of phenolic compounds and their effect on oxidative stress induced by severe physical exercise

**DOI:** 10.1186/s12576-022-00845-1

**Published:** 2022-08-05

**Authors:** Joanna Kruk, Basil Hassan Aboul-Enein, Ewa Duchnik, Mariola Marchlewicz

**Affiliations:** 1 Faculty of Physical Culture and Health, University of Szczecin, Al. Piastów 40b/6, 71-065 Szczecin, Poland; 2 Department of Health Science, Johnson & Wales University, College of Health & Wellness, 8 Abbott Park Place, Providence, RI 02903 USA; 3 London School of Hygiene & Tropical Medicine, Faculty of Public Health and Policy, 15-17, Tavistock Place, London, WC1H 9SH UK; 4 Department of Aesthetic Dermatology, Pomeranian Medical University, Al. Powstańców Wielkopolskich 72, 70-111, Szczecin, Poland; 5 Department of Dermatology and Venereology, Pomeranian Medical University, Siedlecka 2, 72-010, Police, Poland

**Keywords:** Polyphenols, Physical exercise, Oxidative stress, Supplementation, Antioxidant activity, Athletic performance

## Abstract

Extensive research has found strongly increased generation of reactive oxygen species, free radicals, and reactive nitrogen species during acute physical exercise that can lead to oxidative stress (OS) and impair muscle function. Polyphenols (PCs), the most abundant antioxidants in the human diet, are of increasing interest to athletes as antioxidants. Current literature suggests that antioxidants supplementation can effectively modulate these processes. This overview summarizes the actual knowledge of chemical and biomechanical properties of PCs and their impact as supplements on acute exercise-induced OS, inflammation control, and exercise performance. Evidence maintains that PC supplements have high potency to positively impact redox homeostasis and improve skeletal muscle's physiological and physical functions. However, many studies have failed to present improvement in physical performance. Eleven of 15 representative experimental studies reported a reduction of severe exercise-induced OS and inflammation markers or enhancement of total antioxidant capacity; four of eight studies found improvement in exercise performance outcomes. Further studies should be continued to address a safe, optimal PC dosage, supplementation timing during a severe training program in different sports disciplines, and effects on performance response and adaptations of skeletal muscle to exercise.

## Introduction

Studies on the medicinal properties of plant-derived natural phenolic compounds, e.g., polyphenols (PCs) and tea catechins, started in the early twentieth century and the last three decades [1]. Polyphenols having flavonoid structure are the most widely studied category of PCs as an essential component of the human diet. The benefit of these compounds results from antioxidant and anti-inflammatory properties and their impacts on transcription factors and regulation of histone deacetylases activity (an enzyme which regulates proteins expression) as well as from affective protective modulation of cancer initiation and progression, among others [2, 3].

PCs exhibit a wide range of biological activities, such as acting as pigments, anti-inflammatory actions and reducing coronary heart disease, neurodegenerative disease, and cancer risk [4–6]. These compounds are also a subject of great interest within the food sciences due to their potency to preserve food against oxidation and being popular creams' ingredients in skin protection against UV damage [4, 7, 8].

The oxidation–reduction homeostasis and redox signal transduction are critical processes for human health. The disturbance of the redox balance toward oxidation is caused by excessive production of reactive oxygen species (ROS) (superoxide anion radical (O2^**·**^ ®), hydrogen peroxide (H_2_O_2_), hydroxyl radical (HO^**·**^), an electronically excited form of molecular oxygen called singlet oxygen (^1^O_2_), nitrogen monoxide (NO^**·**^), hypochlorous acid (HOCl) and peroxynitrite (ONOO ®)) can induce oxidative stress (OS) [9]. Oxidative stress is "an imbalance between oxidants and antioxidants in favor of the oxidants, leading to disruption of redox signalling and control and/or molecular damage" ([10], p. 181). ROS and RNS are second messengers of intracellular signal transduction, participate in gene expression, exert positive effects on the immune system, regulate angiogenesis, and support relaxation of vascular smooth muscle cells and cellular proliferation [11]. The species are continuously formed during normal metabolic reactions. Their level is regulated by both antioxidant defense systems, enzymatic and non-enzymatic, operating in intracellular and extracellular spaces, preventing or delaying oxidative damage of biomolecules [10]. However, under certain conditions, e.g., insufficient physical activity (PA), extensive or prolonged aerobic or anaerobic exercise with a lack of training, the potency of antioxidant defense systems responsible for keeping cellular levels of ROS/RNS under control may be insufficient. The redox state of cells can be shifted towards oxidizing conditions and cause inflammation (recently summarized in ref. [12]). Therefore, diets rich in vitamins and minerals are essential for intracellular redox regulation [13]. Moreover, there is growing interest in supplementation with natural compounds exhibiting high antioxidative potency. The toxic effects of ROS/RNS are counteracted by enzymatic (e.g., superoxide dismutase, catalase, glutathione reductase) and non-enzymatic (e.g., vitamin C (ascorbic acid), vitamin E, phenolic and PC antioxidants) [14]. The non-enzymatic antioxidants include nutrients not produced by the body but provided by the diet. Phenolics exhibit relatively low bioavailability even after consuming food rich in these compounds [15]. Maximal levels of PCs in the human body were reported at 2–4 h after intake and rapidly decreased. Concentrations of PCs and their metabolites in most humans reach the nanomolar to the low micromolar range [15]. They are much lower than concentrations of small molecule antioxidants, such as the most frequently supplied vitamins C and D, at high micromolar concentrations [15, 16].

In cells, PCs cannot compete with the vital dietary antioxidants such as vitamin C and vitamin E as the potent indirect antioxidants concerning free radicals and ROS scavenging as well as the metal chelators, under normal physiological conditions. However, PC concentrations are high enough to exert activity at enzymes, receptors, or transcription factors, under in vivo conditions [17].

Most PCs are amphiphilic. Thus, they exhibit both hydrophilic and lipophilic properties, which determine their adsorption on the cell membrane surface and/or insertion into the lipid bilayer and interaction with the hydrophobic chains of lipids, followed by scavenging free radicals formed during lipid peroxidation. Consequently, PCs can protect cell membranes and their components against oxidative damage and modify the activity of membrane-associated proteins [16]. The PC–protein interactions include interactions of PCs with enzymes, and transcription factors estrogen receptors, among others [15]. Evidence underlines their essential role in amplifying the antioxidant defense system and stimulating the expression of antioxidant enzymes through the extracellular signal-regulated kinase/the nuclear transcription factor–erythroid 2-related factor 2, (ERK/Nrf2) pathways signalling in the antioxidant activity of some PCs [18]. Specifically, PC antioxidants decrease ROS formation mainly by inhibiting enzymes involved in their generation and upregulation or protection of the antioxidant system. In turn, vitamin C is a reducing hydrophilic antioxidant, playing a pivotal role in quenching various ROS/RNS and acts as an electron donor in non-enzymatic reactions; the compound is considered a potent water-soluble free radical scavenger. Vitamin C is also a substrate for ascorbate peroxidase [17]. Vitamin E presents a set of eight compounds: four tocopherols and four tocotrienols. The compound is fat-soluble, and one of the tocopherols-α-tocopherol is the highest activity in humans. Vitamin E is a lipophilic compound known as the most potent antioxidant, protecting cellular membranes against free radicals (e.g., lipid-peroxyl radicals) formed during lipid peroxidation. Both vitamins C and E function mainly as scavengers of free radicals, while PCs show antioxidant and anti-inflammatory properties above all through interactions with cell components [19].

Grapes, soy, green tea and chocolate are the most consumed natural food products rich in PCs with strong antioxidant potency [4, 20, 21]. Growing evidence has shown that oral supplementation with PCs in rats and athletes enhances the endogenous antioxidative defense system and protects against OS [22, 23]. It has also been reported that supplementation with an N-acetylcysteine donor (NAC) may enhance the cell adaptation to physical training by increasing glutathione (GSH) synthesis [24, 25]. However, there is a limited number of studies on the effect of PC supplementation on exercise-induced OS in athletes. The phenolic-rich fruits and vegetables in a diet contribute to the athlete’s antioxidant cellular status. Additional supplementation with a high dose of PCs, such as other antioxidants, may cause side effects. The literature has documented that PCs exhibit toxic pro-oxidative effects at high concentrations [23, 26–30]. Several clinical studies showed that taking antioxidant supplements, including vitamins A, C, and E, as well as β-carotene and PCs, can accelerate tumorigenesis [31, 32].

Moreover, it has also been demonstrated that exogenous antioxidants at high doses can display double-edged effects on inflammation [33]. Hence, a need has appeared to measure OS levels in athletes and special dietary supplements by type, safe dose, and intake duration. Evidence has reported several diverse measure techniques for detecting ROS [34, 35] and their usage to evaluate the effectiveness of antioxidants in biological systems, e.g., by measuring total antioxidant capacity (TAC) and levels of OS biomarkers. This review presents the extent of knowledge on the antioxidant capacity of phenolic antioxidants and their supplementation concerning possible benefits for athletes in the contest OS and inflammation induced by high-intensity endurance exercise. We have also attempted to provide insight into the potential mechanisms, particularly the antioxidant–endurance exercise-induced OS interactions, focusing on current evidence. In addition, we discussed the effect of PC supplementation on endurance exercise performance and the role of ROS in skeletal muscle adaptations to physical exercise (PE). In addition, most applied techniques for determining OS markers and antioxidant capacity are reviewed.

### Sources and chemical structure of phenolic compounds

**Fig. 1:**
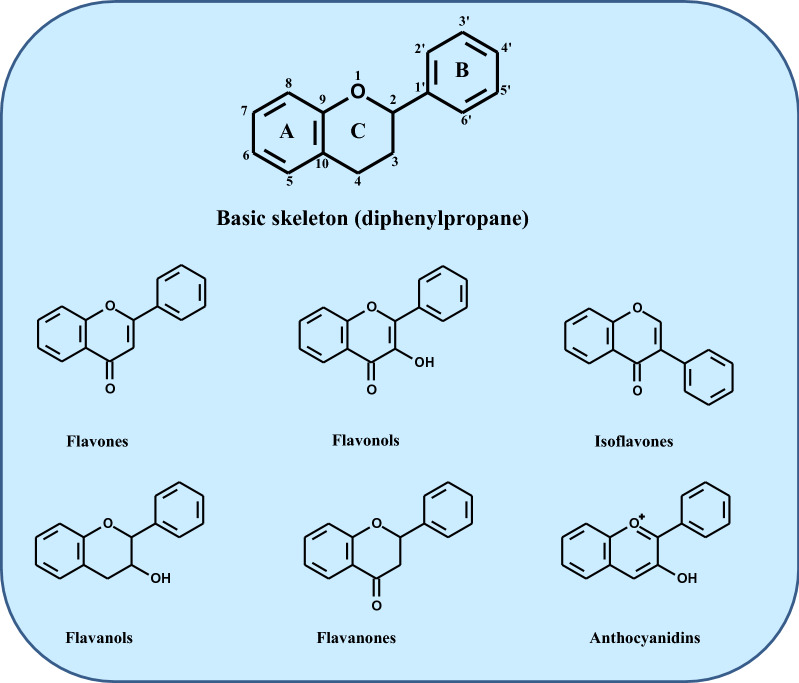
Chemical structures of flavonoids and their main subclasses

Phenolic compounds comprise a multi-numerical and diverse family of natural antioxidants present in most fruits, vegetables, seeds, plant-derived beverages (tea, juice), and other plant-based foods [36–38]. Evidence has reported the list of 100 dietary products which are richest in PCs, finding their concentrations ranging from 15,000 mg/100 g in cloves to 10 mg/100 mL in red wine, with as many as 89 foods and beverages containing more than 1 mg of total PCs/serving [39]. It is estimated that the average intake of PCs from phenolic-rich foods is about 1.0 g/day. Although the daily consumption of the compounds is relatively high, their bioavailability is low. According to the evidence, PC concentration in human plasma rarely is higher than 1 μM in individuals even consuming large amounts of vegetables and fruits or taking them as a diet supplement [4, 40]. PCs possess one or more hydroxyl groups (-HO) attached to a benzene ring [15, 36]. Based on chemical structure, five main classes of phenolics are distinguished among ten or more types, i.e., phenolic acids (e.g., caffeic acid, gallic acid), flavonoids, stilbenes (e.g., resveratrol), lignins (cross-linked phenolic polymers), and condensed tanins (large molecules derived from phenolic acids) [7, 8, 37, 38, 41]. Flavonoids are the most widespread PC class (∼80% of all PC compounds). This class of PCs has a chromane-type skeleton with two aromatic rings (A- and B-rings) linked through three carbon atoms, forming heterocyclic ring C containing an oxygen atom (Fig. 1) [8, 36, 37, 42, 43]. The flavonoids have six main subclasses: flavones, flavonols, isoflavones, flavanols, flavanones and anthocyanidins (Fig. 1).

All these compounds exhibit the characteristic C_6_–C_3_–C_6_ carbon skeleton. Evidence showed that flavanols, flavonols and anthocyanins dominate in terms of presence in land plants.

*Flavones* structure shows a double bond between C_2_ and C_3_ and a carbonyl group (C = 0) on the C ring at C_4_. For example, luteolin, a potent antioxidant in many vegetables, fruits and olives, possesses four -OH groups, two groups situated on the A-ring at C_5_ and C_7_, and two -OH groups at 3’ and 4’ position of the B-ring (5 = 7 = 3’ = 4’ = OH).

*Flavonols* are similar in structure to flavones, but one atom of hydrogen (H) at the C_3_ position of the C ring is replaced by the –OH group. The primary dietary representative of flavonols is quercetin (5 = 7 = 3’ = 4’ = OH), present in many foods, e.g., apples, onion, tea, and tomatoes.

Unlike flavones and flavonols, *Flavanones* have the C ring saturated and easily undergo hydroxylation. Naringenin (5 = 7 = 4’ = OH) is the dominant representant of this subgroup and is present, e.g., in herbs and tea.

*Flavanols* (flavan-3-ols) are similar in structure to the flavanones, but the -OH group replaces the H atom at the C_3_ position of the C ring. Catechin (5 = 7 = 3’ = 4’ = OH) and epicatechin, found at high concentrations, e.g., in apples, cherries, cacao/dark chocolate, are exemplary flavanols.

*Isoflavones*, this flavonoid subgroup, have the B ring attached to the C ring at the C_3_ position. Thus, they are structurally like estradiol. Daidzein (7 = 4’ = OH) and genistein (5 = 7 = 4’ = OH), prevalent dietary supplements present in high concentrations in soy, are the isoflavones examples [44].

*Anthocyanidins* are pigments occurring in the cation form (flavylium cation) in flowers, fruits, vegetables, and seeds, the only group among flavonoids responsible for the colour of the plants. All free rings A, B, and C are aromatic; these compounds have the -HO group at the C_3_ position of the C ring, stabilizing the flavylium cation. The most common anthocyanins found in plants have the 3-glycoside chemical structure [45, 46]. Substitution of one phenol ring in anthocyanidin with glycosides leads to the formation of anthocyanin [47]. The presence of the phenolic –OH group in the chemical structure of flavones, flavonols and anthocyanidins enables the generation of O-glycosides or C-glycosides with sugar/s [36, 42]. For example, the conjugation of flavonol quercetin with disaccharide rutinose (position C_3_ of the C ring) forms quercetin− 3–0-rutinoside (rutin), among others, in citrus fruits. Comprehensive reviews are available for more detailed data [1, 36, 42, 48].

### Chemical and biological features of polyphenols

Polyphenols demonstrate considerable ability to combat and protect against the effects of OS. Evidence has revealed that PCs offer protection against several diseases (cardiovascular, neurological) and lower the risk of chronic diseases, e.g., inflammation, diabetes, hypertension, and cancer [3, 4, 6, 15, 23, 49, 50]. Two potential protective mechanisms of phenolic action have been proposed: non-specific interactions and specific [15]. The non-specific actions are based on free radical scavenging capacity and metal chelation, owing to the presence of phenolic groups and the presence of 2–3 double bond and a 4-keto group in ring C (direct effects). In turn, the specific mechanisms are linked with structural and conformational properties of PCs and interactions with biomolecules, e.g., proteins or specific membrane components, such as lipids [15].

### Antioxidant properties of phenolic compounds

Evidence has shown that PCs are recognized as active antioxidants even at low concentrations, although most of the evidence of antioxidant capacity originates from in vitro studies. Considering their redox properties and the ability to self-oxidize at higher concentrations, PCs can also show prooxidant activities in vivo under certain conditions, such as high doses supplementation, high concentration of transition metal (e.g., Cu, Fe, Zn) ions, alkaline pH, presence of molecular oxygen, [23, 26–30, 51]. For example, the increased concentration of serum transaminase and bilirubin, inflammation and necrosis were observed during biopsies in some individuals taking the high dose (700–2100 mg/day) of green tea supplements [29]. In addition, intra-individual susceptibility to the toxic effect of PCs remains dependent on the metabolism and the supplement bioavailability. Experimental studies have presented that caffeic acid exhibits prooxidant activity at concentrations 200 μM, quercetin—> 100 μM, naringenin—> 200 μM, epigallocatechin-3-gallate—at 100–500 μM [29]. Evidence underlines that diet daily PC intake shows no prooxidant activity in tissues [26, 28, 43, 51]. There is a clear consensus in the literature that exogenous antioxidants, such as food constituents, such as antioxidant enzymes, neutralize free radicals and ROS/RNS and remove or repair damaged biomolecules [52]. Free radicals, e.g., O_2_^**·**^‾, HO^**·**^, peroxyl radical (ROO^**·**^), NO^**·**^ as well as non-radical species formed from molecular oxygen, such as H_2_O_2_, HOCl are continuously produced at low concentrations during normal physiological processes. The physiological level of ROS/RNS is essential for immune system function. They play a crucial role in cell signal transduction, inflammatory responses, gene expression, regulation of smooth muscle, destruction of pathogens in cells by nicotinamide adenine dinucleotide phosphate (NADPH) oxidase during tissue infections, diffusing through membranes of pathogens and damaging their DNA and other biomolecules [9, 53]. Antioxidant systems control amounts of these species to maintain redox homeostasis [9]. However, the O_2_^**·**^‾, H_2_O_2_, and NO^**·**^ species generated at high concentrations can be toxic for cells, producing several more reactive species, i.e., HO^**·**^, ^1^O_2_ and peroxynitrite (ONOO‾). For example, O_2_^**·**^‾ reacts easily with NO^**·**^ generating ONOO‾ and HO^**·**^ as follows [54]:

1 $${\text{O}}_{2}^{ {\boldsymbol{\cdot}} - } + {\text{NO}}^{ {\boldsymbol{\cdot}} } \to {\text{ONOO}}^{ - }$$

followed by formation of HO^**·**^ [55]. Another important reaction generating HO^**·**^ in vivo is the Fenton-like reaction in which H_2_O_2_ is reduced by transition metal ions (Me^n^), e.g., Fe(II), Cu(I) [56]:

2 $${\text{Me}}^{{\text{n}}} + {\text{ H}}_{{2}} {\text{O}}_{{2}} + {\text{ H}}^{ + } \to {\text{ Me}}^{{{\text{n}} + {1}}} + {\text{ HO}}^{ {\boldsymbol{\cdot}} } + {\text{ H}}_{{2}} {\text{O}}$$

In turn, hypochlorite ion (OCl‾) relative quickly reacts with H_2_O_2_, forming another powerful oxidant, ^1^O_2_ [57]. These species are produced in the cell in excess. They can cause oxidative damage to proteins, lipids, DNA, RNA, and polysaccharides under conditions when levels of the oxidants exceed the capacity of endogenous and exogenous antioxidant defense systems and potency of repair mechanisms, resulting in disturbance of redox homeostasis with the consequence of OS [10]. Long-term OS has been reported to contribute to inflammatory diseases, cardiovascular diseases, diabetes mellitus, neurodegenerative diseases, reproductive system diseases, cancer, and ageing [58–61].

In vitro, evidence showed that PCs have strong antioxidant power due to their free radical and ROS/RNS deactivation capacity and are effective metal chelators, e.g., Fe(III) [1, 36, 62, 63]. These compounds can donate H-atom to a free radical (R^**·**^) through the H-atom transfer mechanism, forming peroxyl radical (PCO^**·**^)

3 $${\text{PC}}\left( {{\text{OH}}} \right) + {\text{R}}^{ {\boldsymbol{\cdot}} } \to {\text{ PCO}}^{ {\boldsymbol{\cdot}} } + {\text{RH}}$$

acting as chain-breaking agents of lipids oxidation and/or peroxidation [8]. Experimental studies showed that PCO radicals might undergo recombination (2PCO^**·**^ → PCO–OPC), forming non-reactive dimers or acting as a resonating compound and can be stabilized by resonance effect and/or to form an intermolecular hydrogen bonding [1, 36]. The stability of PCO^**·**^ determines the antioxidant efficiency of the PC. This process is strongly dependent on the PC chemical structure, especially on the presence of additional -OH groups, their number and reactive position, influencing the delocalization of an electron over the benzene ring.

In vitro evidence has demonstrated that the PCs which have a catechol moiety in the B ring, a double bond between C_2_ and C_3_ in the C ring and, in addition, they have HO groups linked with the A ring at C_3_ and C_5_ positions (Fig. 1) exhibit high antioxidant efficiency. The presence of the catechol structure of the B ring is crucial in this respect [1]. The hydrogen atom transfer efficiency negatively correlates with the O–H phenolic bond strength. With a weaker bond, the antioxidant power is higher. Electron-donating substituents at the ortho and/or para positions lower the phenolic O–H bond dissociation energy. Studies on the association between the chemical structure of PCs and their antioxidant power showed that flavonols exhibit higher antioxidant power than flavones [1, 42]. Both experimental studies and quantum chemical calculations showed that the H-atom transfer is linked with both atom or electron transfer reactions [62]. In the single electron transfer, an electron is transferred from PC to free radical (R^**·**^), forming a chemically stable radical cation PCO^**·**+^, and R^**·**^ is reduced to an anionic form (R‾) [1]:

4 $${\text{PC}}\left( {{\text{OH}}} \right) + {\text{R}}^{ {\boldsymbol{\cdot}} } \to {\text{ PCO}}^{ {\boldsymbol{\cdot}} + } + {\text{R}}^{ - }$$

In this process, the antioxidant power is dependent on an ionization potential. The lower ionization potential means a higher probability of the electron transfer to a radical [1, 62]. The process may be two-step depending on pH, and the interaction of PCO^**·**+^ with R‾ is possible [62]:

5 $${\text{PPO}}^{ {\boldsymbol{\cdot}} + } + {\text{ R}}^{ - } \to {\text{PCO}}^{ {\boldsymbol{\cdot}} } + {\text{ RH}}$$

Other structural feature of PCs important for their antioxidant potential includes an ability to chelate transition metal ions (e.g., Fe, Cu) responsible for free radicals’ production [64]. The presence of 3- or 5-hydroxy group and unsaturation in the C ring and the carbonyl group at the 4-position of this ring (Fig. 1) are metal ions sensitive complexing domains. In addition, the dihydroxylated B ring in certain types of PCs is a second metal ion-binding site [63]. However, due to a low concentration of PCs in human tissues, their ability as inhibitors of free radicals and ROS/RNS as well as effective metal chelators is suggested to be limited. That results from a large amount of redox reactive metal ions and/or a type of compartments, where concentrations of these compounds are high, e.g., in the gastrointestinal tract [15].

### Biological activity of phenolic compounds

The spectrum of biological activity of PCs is broad. Briefly, the nonspecific protective mechanisms include interactions of PCs with membranes due to their both hydrophobic and hydrophilic interactions. This property allows most of the PCs to be placed at different membrane levels, i.e., on the polar head groups of phospholipids, forming the hydrogen bonds, inserting into lipid bilayers, and interacting with the hydrophobic part of the lipids chain. This way, some PCs can protect membranes and their components from oxidative damage.

The specific mechanisms (indirect effects) responsible for the biological effects of PCs are also related to interactions with proteins and depend on proteins' functions and metabolites of their biotransformation [65]. This type of mechanism includes interactions with enzymes which are involved in inflammation, such as phospholipases A2 (PLA_2_), cyclo-oxygenase enzymes 1 and 2 (COX1/2), and 5-lipoxygenase (LOX), followed by a decrease of O_2_^**·**^‾ level and NO^**·**^ availability, and regulation of blood pressure. Another mechanism includes modulation of redox-sensitive transcription factors, e.g., the nuclear factor kappa B (NF-κB) (involved in intracellular signalling cascades) and transcription factor activator protein (AP-1) or in an interaction with estrogen receptors, acting as estrogen agonist or antagonist, due to isoflavones structural similarity to estrogens [66]. Some studies reported that PCs at physiologic doses could suppress markers of OS, i.e., the secondary products of inflammation response to external stressors [23, 67]. Tyrosine and serine–threonine protein kinases are the enzymes that are mainly deactivated by PCs, limiting the production and release of ROS/RNS and proinflammatory cytokinesis [23]. The anti-inflammatory action and immune modulation of PCs reported by many epidemiological and experimental studies have shown that these natural compounds can influence the immune cells population and modulate tissue balance between proinflammatory cytokines (e.g., IL-1β, IL-2, IL-6, IL-8, TNF-α) and anti-inflammatory cytokines (e.g., IL-4, IL-10, TGF-β) [67]. Animal and epidemiological studies have reported that PCs inhibit tumour activity by reducing cell proliferation and survival, mutagenesis, angiogenesis, apoptosis, and leucocyte immobilization [3, 6, 58, 67].

**Fig. 2:**
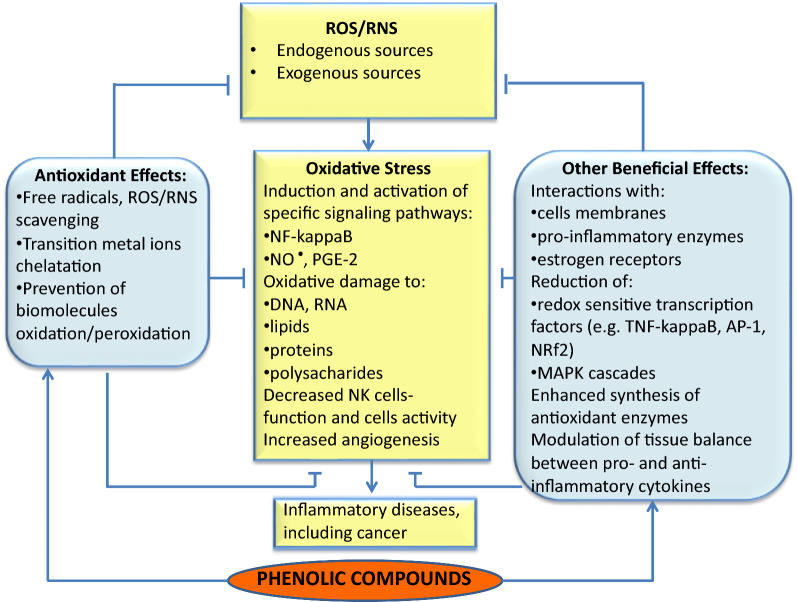
Simplified scheme presenting oxidative stress consequences and the beneficial effects of phenolic compounds

Furthermore, inhibition of the mutant p53 protein, heat shock proteins, Ras protein expression, and cell cycle arrest in proliferating lymphoid cells has been hypothesized to lower cancer risk. Prevention against overaction of Nrf2 and NF-κB activation by PCs in cells, followed by the influence on the mitogen-activated protein kinase (MAPK) signal transduction and phosphatidylinositol 3-kinase (P13K) pathways, has been suggested as essential functions in the proliferation of cancer cells [6, 15]. In addition, PCs induce the cell defense systems by enhancing the synthesis of endogenous antioxidant enzymes [22, 23, 67]. (For more information on the antitumor activity of PCs and their signalling pathways, see Refs 6, 67–69). Increasing evidence available from in vitro and in vivo studies has reported that PCs as bioactive molecules can regulate muscle homeostasis, prevent catabolism, and enhance their anabolism due to their anti-inflammatory and antioxidant function [19]. However, the biological mechanisms linked with the association between antioxidant and anti-inflammatory properties of PCs and human health benefits are still the subject of discussion. These beneficial health abilities of PCs in coping with excessive ROS production and developing OS, and preventing inflammation are summarized in Fig. 2. ROS/RNS are generated from both endogenous sources (e.g., mitochondria, peroxisomes, microsomal cytochrome P450, metabolism of xenobiotics) and exogenous sources (e.g., ultraviolet radiation, inflammatory cells, pathogens, toxin exposure, strenuous/exhaustive PE) [69]. Acute inflammation is mediated through activation of the immune system that recognizes endotoxins and uses ROS. The process is usually beneficial, whereas chronic inflammation can predispose individuals to several diseases due to excess ROS/RNS [70]. When inflammation begins, most cells and leukocytes are focused on the site of damage, leading to a "respiratory burst" caused by increased oxygen uptake and excessive ROS production. This process is enhanced as specific immune system cells secrete signalling molecules, such as metabolites of arachidonic acid, cytokines, and chemokines. The biomolecules recruit inflammatory cells to the site of damage, and the production of ROS/RNS is enhanced [71]. Proinflammatory cytokines are involved in the upregulation of inflammation through activating signalling cascades and induction of changes in transcription factors (e.g., NF-κB, STAT3, HIF-1α, Nrf2) that modulate a wide range of cellular functions. Furthermore, induction of COX-2, iNOS, TNF-α, IL-1, IL-6 and expression of mRNAs have been suggested to play a crucial role in OS-induced inflammation [71]. The continuation of OS can lead to chronic inflammation, which can mediate inflammatory diseases [71, 72].

### Exercise and oxidative stress

Strong evidence exists on the beneficial effect of regular moderate-to-vigorous PA/PE on human health [61, 73–79]. The benefits are achieved through increases of insulin sensitivity [76]; regulation of energy expenditure, insulin-like growth factor-1 (IGF-1) and insulin-like growth factor binding proteins (IGFBPs) balance; improvement of lipid and carbohydrate metabolism [77]; reduction of endothelial dysfunction [78, 79]; increase of catecholamines secretion (the compounds restore the disturbed redox balance and improve insulin sensitivity); reduction of visceral adipose tissue; activation of redox-sensitive signal transduction and enhancement of expression of genes responsible for the production of antioxidant enzymes (e.g., superoxide dismutase (SOD), catalase, and glutathione peroxidase (GPx)) in muscles [71, 80–82]; mitochondrial biogenesis; reduction of inflammation, changes in metabolic and sex hormones metabolism; enhancement of immune system response, and up-regulating resistance to OS [40, 83]. Evidence has maintained that PA of moderate to vigorous intensity produces ROS/RNS at concentrations which may be required for the proper signalling and an adaptation to excessive species production in response to training [84]. Moreover, epidemiological and clinical research findings have shown that PE can decrease the risk of occurrence of more than 35 human health disorders [85]. However, PE-induced health benefits can be limited by the overproduction of ROS/RNS. Excessive ROS/RNS can cause an extensive alteration in redox homeostasis, damage biomolecules and decrease skeletal muscle force, overtraining, muscle injury, and illness [86–88]. A considerable number of studies have reported that not only a sedentary lifestyle but also short-term exercise of high intensity or severe aerobic or anaerobic PE not preceded by training can primarily increase ROS/RNS production, elevating the inflammatory processes in the cells and contribute to OS [89–91]. For example, an experimental study by Diaba-Nuhoho et al. [91] observed significant increases in markers of OS, such as malondialdehyde (MDA), uric acid, and SOD which were positively correlated with vigorous PE (*r* = 0.715, *r* = 0.512, and *r* = 0.810, respectively); MDA also was found to be correlated with PE of moderate intensity (*r* = 0.841). Activation of the xanthine oxidase pathway, NADPH oxidases, and mitochondria cellular respiration are reported as ROS' primary sources [73]. Extensive PE is linked with increased uptake of molecular oxygen compared to oxygen uptake at the resting state, which may contribute to the overproduction of ROS/RNS in skeletal muscle [73, 91–93]. It has been suggested that the contribution of severe PE in the development of OS is dependent on age, gender, exercise type, intensity and duration of the training, and exposure of individuals to environmental factors (food, alcohol consumption, cigarette smoke, air pollution, pesticides, heavy metals, plastics, exposition to UV radiation) [94, 95], and the rest redox individual's homeostasis [92]. However, several studies have suggested that regular training does not involve OS in contracting muscles, which is in accordance with the hormesis theory [12, 24, 96]. Findings provided strong evidence that aerobic and anaerobic PE increases ROS/RNS production rates, exerting the effect on the cellular redox homeostasis [12, 89]. Independently on exercise mode and intensity, an acute bout of PE resulting in ≥ 50 VO_2_ max increases ROS/RNS levels above physiological level. The level of ROS/RNS depends on many factors (e.g., exercise characteristics and duration, age, gender, training status, availability of protein thiols to sites of the species generation, diet) and maybe or not accompanied by acute OS [24, 73, 75, 89, 97]. ROS/RNS generated at increased concentrations under stressful conditions exhibit positive and negative effects on the human body. Evidence has suggested that acute endurance exercise can cause OS [97], while the regular exercise of moderate intensity (50– < 60 VO_2_ max) regulates the levels of ROS/RNS, acting as intracellular signalling agents; it can start the exercise adaptation to OS, due to upregulation of antioxidant defenses and mitochondrial biogenesis [75, 82]. The health benefits of PA/PE are specific to the mode of exercise performed in adaptive responses to free radicals and ROS/RNS generated in skeletal muscle. Regular resistance training of low-load stimulates the myofibrillar proteins. It increases hypertrophy of the muscle cells and maximal muscle strength without compromising muscle myofibril accretion, while endurance and high-intensity training stimulate mitochondrial muscle biogenesis, increase muscle fibres' respiratory capacity and improve their endurance [98]. Evidence underlines that endurance exercise is needed to drive mitochondrial adaptations in skeletal muscles, whereas resistance exercise is required to induce myofibrillar protein adaptations [98, 99]. A review by Hughes et al. [100] has suggested that increased intensity of PE, such as sprint interval training and high-intensity interval training, enhances mitochondrial cell number within skeletal cells. It increases the respiratory capacity of muscle fibres and maintains that genes can determine individuals' response to training. In addition, prolonged endurance exercise of low intensity but high volume is accompanied by increased mitochondrial content within skeletal muscle [100].

Recently, concern has been raised about the diverse effects of one of several cytokines produced by human cells, i.e., cytokine IL-6. Exercise induces IL-6 production in skeletal muscle in much larger concentrations than other cytokines [101]. Cytokine IL-6 exhibits multifunctional action, such as metabolic functions (e.g., promotes catabolism and anabolism, induces lipolysis, glycolysis, free fatty acids secretion, glucose release during exercise), immune functions (e.g., proinflammatory and anti-inflammatory actions), and musculoskeletal functions (catabolic and anabolic effects). Thus, it can have both beneficial and harmful effects on human health. A recent review by Kistner et al. [101] addressed the properties of this cytokine depending on exercise duration. Research findings have shown that IL-6 is released from skeletal muscle mitochondria in response to a deficit of energy. The short-lasting excess of IL-6 arising during bouts of exercise releases somatic energy through upregulation of lipolysis and gluconeogenesis. It enhances energy uptake by muscle and transiently down-regulates the immune system activity by a decrease of proinflammatory cytokines (TNF-α and IL-1β) production. In this respect, there is a growing consensus that IL-6, when released by skeletal muscle during PE, has anti-inflammatory properties. In addition, IL-6 increases the production of anti-inflammatory cytokines (IL-10 and IL-Ra), apoptosis, insulin secretion, and muscle sensitivity. In turn, the chronic release of cytokine IL-6, e.g., during prolonged PA/PE, activates the immune system, exerts a catabolic effect on muscles, i.e., mediates muscle proteins degradation and reduces their synthesis, contributes to insulin resistance and adipose, increases the blood level of glucose, accumulates lactic acid, and increases ROS/RNS generation along with muscle glycogen depletion and reduction of energy stores in the muscle [101]. Due to modulation signalling pathways, inflammation and mitochondrial dysfunction are essential contributors to muscle atrophy [19].

According to Radak et al.’s [93], the hormesis theory extended to an adaptation of the organism to PE exposure shows that small amounts of exercise-induced ROS exert an adaptive effect on cells. In contrast, high amounts of stressors may be damaging to cells. The beneficial effect occurs when the exercise dose is within a specific range of the dose–response curve (ROS production–physiological response) having the bell shape and is followed by a rest period. On the other hand, when PE is too severe or not followed by the rest, the potency of the endogenous antioxidant defense systems is insufficient to destroy large amounts of ROS/RNS. Under these conditions, excessive production of the toxic species or a defect in their removal can result in inflammation and oxidative damage of nucleic acids, lipids carbohydrates, consequently leading to DNA damage, ageing and degenerative diseases, including potential cancer development, as summarized in Fig. 2 [10, 12, 60, 102].

Evidence exists for a decrease in total antioxidant status (TAS), increased lipid peroxidation markers and inflammatory cytokines and muscle damage in athletes participating in marathons or ultramarathons despite the training compared to sedentary individuals [24, 103]. PLA_2_ and two isoforms of NADPH oxidases: NOX2 and NOX4, with a minor mitochondria contribution, are considered key sources of ROS in contracting skeletal muscle [92]. PLA_2_ can promote ROS generation in the cytosol and mitochondria during the rest state and exercise. In turn, NOX2 has been suggested as a ROS generator in contracting skeletal muscles due to activation of NADPH oxidases and NOX4 as a contributor to forming the species in muscle fibres [92]. To date, it has been maintained that the production of ROS by exercise is essential in the activation of the transcription factor NF-κB signalling pathway, known for participation in the progression of certain cancers as well as in induction of peroxisome proliferator-activated receptor gamma coactivator 1-alpha (PGC-1α). PGC-1α is involved in regulating cellular metabolism and is used by invasive cancer to enhance oxidative phosphorylation and mitochondrial biogenesis [92, 104]. Evidence has shown increased plasma inflammatory levels of cytokines and OS markers, e.g., TNF-α, IL-6, CRP, linked with severe/exhaustive PE [105]. Although research consensus indicates that PE enhances the generation of ROS, there is a discussion about whether exhaustive PE produces high enough concentrations of ROS to lower the effectiveness of antioxidant systems and to cause OS and oxidative damage to the trained muscles [74]. This discussion and many inconsistencies in the PE–OS association seen in literature originate from the methodical difficulties for the direct and quantitative detection of free radicals and other ROS/RNS. It results from their short lifetime, instability of the oxidation products (e.g., lipid hydroperoxides in the presence of transition metal ions), diversity of PE-training protocols, lack of noninvasive measurement assays, and specificity of the measured OS and inflammation markers.

### Determination of oxidative stress and antioxidant capacity

Several reports have demonstrated training-induced up-regulation of endogenous antioxidants, e.g., SOD activity [76, 81, 106]. On the other hand, many studies have reported the effect of acute PE as decreased TAS of plasma and increased level of OS markers [91, 107]. Evidence exists that a diet rich in antioxidants and some antioxidants intake may limit biomolecules' oxidative damage, improve redox balance in living organisms, and reduce OS induced by severe PE [13, 89, 91, 107].

The basic problem in detecting free radicals and non-radical ROS/RNS in the biological system is their short lifetime and high reactivity [11]. Because ROS/RNS starts OS, a direct measure of their cellular concentrations is one of the approaches to evaluating OS in cells and tissues [108]. The electron spin resonance (ESR) spectroscopy, the accepted method for giving information about the level of free radical, has limited use in in vivo systems because of the high absorption of microwave energy in aqueous solutions. Therefore, measurements are usually carried out at low temperature or using nitrone spin traps (e.g., 5,5-dimethyl-1-pyrroline-1-oxide (DMPO), N-tert-butyl-α-phenylnitrone (TMPO)) [109, 110]. In addition, a tissue sample preparation process may be accompanied by ROS production. It shows that the direct measure of ROS/RNS levels using expensive ESR apparatus is not suitable for measuring OS in skeletal muscle. The activity of antioxidant enzymes, e.g., SOD, GPx and concentrations of nonenzymatic antioxidants determine the level of OS in tissue. An important marker of tissue reduction/oxidation homeostasis is the ratio of reduced glutathione (GSH) to oxidized glutathione (GSSG) [9]. The most commonly preliminary measure of changes in GSH levels in plasma and tissues involved by PE has been used for a long time [109] to obtain information on free radicals and antioxidants levels and cellular redox homeostasis. Direct useful probes for cellular OS measure include chemiluminescent luminol (5-amino-2,3-dihydro-1,4-phthalazine–dione) and lucigenin (10,10’-dimethyl-9,9',-biacridinum dinitrate) probes, acting as enhancers of light emission arising in chemical reactions. This process is accompanied by the formation of electronically excited products, which release their energy during decay to a ground state [110]. The light emission is observed, e.g., during oxidation of luminol with O_2_^**·**^‾ and oxidation of lucigenin with O_2_^**·**^‾, H_2_O_2_ or ^1^O_2_. However, the lucigenin- or luminol-enhanced chemiluminescence techniques are usually unsuitable for exercise studies due to the redox cycling properties of these chemiluminescence probes [111]. Another direct approach in the evaluation of OS in living cells is the usage of fluorescent-dye-based assays [108, 112], e.g., 5-(or-6)-carboxy-2',7’-dichlorofluorescein diacetate (DCFDA—cellular ROS assay kit). The method uses fluorescence spectrometry and cell suspension and is based on DCFDA diffusion into the cells, wherein the reaction with H_2_O_2_ transforms to 2',7’-dichlorofluorescein. The last compound emits light after excitation, of which intensity is positively correlated with levels of H_2_O_2_, HO^**·**^, ROO^**·**^ in the cells. Evidence showed that another fluorescent probe, dihydroethidium, in the reaction with O_2_^**·**^‾ is oxidized to ethidium bromide exhibiting light emission after excitation proportional to the cellular O_2_^**·**^‾ concentration.

In 2014, La Favor et al. [111] presented a novel technique based on microdialysis and a specific fluorogenic substrate (Amplex^®^ UltraRed) dichlorodihydrofluorescein that can be used for the detection of combined H_2_O_2_ and O_2_^**·**^‾ in vivo in human skeletal muscle safely and with minimal invasiveness. Briefly, Amplex^®^ UltraRed reagent and horseradish peroxidase were perfused through microdialysis probes and inserted into the vastus lateralis of healthy volunteers. In the presence of peroxidase, this molecular probe reacts with H_2_O_2_ in the extracellular environment, forming the highly fluorescent resorufin inside the microdialysis probe. The fluorescence signal was detected at 590 nm (λ exc = 510 nm), and the relative intensity of the signal was converted to H_2_O_2_ concentration based on the H_2_O_2_ standard curve. These authors concluded that this technique does not give information on the absolute concentration of H_2_O_2_ or O_2_^**·**^‾, but may be used to compare the interstitial ROS levels between groups or efficacies of oral antioxidant supplementation. In addition, they also implemented the microdialysis technique to measure levels of H_2_O_2_ and O_2_^**·**^‾ in the extracellular environment of various rodent tissues and the mitochondrial release of ROS in fibres [113].

The chemical properties of biological oxidants cause indirect measures of redox homeostasis via detection of suitable biomarkers of biomolecules damage induced by ROS/RNS are more utilized than a direct measurement of their levels [97, 112]. It is important to note that knowledge of the participation of OS in cellular and tissue damage and the pathology of inflammatory disease is mainly based on animal models [112]. Biomarkers are an essential tool in both the OS evaluation as well as in health prevention by antioxidants. Several analytical assays based on the detection of ROS/RNS-induced modifications of biomolecules, such as spectrometry, gas and liquid chromatography, spectrophotometry, colorimetry, enzyme-linked immunosorbent assay kits (ELISA), fluorimetry have been widely used to assess the products of oxidative damage of lipids, peptides, carbohydrates and oxidative modification of DNA and RNA as OS markers [91, 108, 114]. For example, alkanes, malonaldehyde (MDA), and F-isoprostanes are utilized as markers in lipid peroxidation measurement techniques. In turn, detections of products containing carbonyl groups, peptides nitrated by ONOO‾, products of fragmentation of peptide chain and aggregation products arising during cross-linked reactions, as well as a change of electric charge density have been applied to measure oxidative damage to proteins. Two methods are mainly used for protein detection, e.g., immunohistochemistry and western blotting [87].

Furthermore, products of guanosine oxidation by HO^**·**^, such as 8-hydroxydeoxyguanosine (8-OHdG) and 8-hydroxyguanosine, are the most popular markers of DNA/RNA damage, respectively. However, cell count and viability affect the evaluation of ROS outside the cells [108]. Alternative to measuring markers of biomolecule damage, OS is also characterized by the total oxidant status (TOS) and the total antioxidant status (TAS), using the methods developed by Erel [115, 116]. Sample antioxidant capacity is usually expressed as millimoles per litre of Trolox equivalents. Another parameter, the oxidative stress index (OSI), defined as the ratio of TOS to TAS, more precisely characterizes the overall OS status in the body. The TAS and TAC assays are applied to measure the redox state of biological fluids, cellular supernatant, and animal and plant tissues. They use selective colorimetric assay kits and the colorimetric technique. Effective supplementation of antioxidants should be proceeded by monitoring TAS; the assay provides information about the overall antioxidant status of an individual’s body. In turn, the total antioxidant capacity (TAC) assay gives information about the antioxidant ability to scavenge free radicals. Evidence has suggested that OXY-SCORE or Oxidative-Index calculated by subtracting the TAC from the OSI is more informative in evaluating redox balance in the exercise conditions [112].

However, indirect assessment of redox status in blood via the OS markers is invasive due to the blood collection from a vein. The analysis requires expensive apparatus, which is impractical for assessing OS in athletes. A point-of-care assay that uses portable blood analyzers is more helpful in this regard. The method is less invasive, because it allows the acquisition of blood samples from a fingertip-or-earlobe-pick [117]. In this method, two tests: Free Oxygen Radical Test (FORT) and Free Oxygen Radical Defence (FORD), developed by Pavlatou et al. are used [118]. These tests helped measure hydroperoxide levels in the whole blood sample and antioxidant ability, mainly ascorbic acid, GSH, albumin, and Trolox. The assay has been used in clinical studies and for the athletes' response to acute exercise to determine the disturbance of redox balance at rest, obtain necessary information regarding an athlete's health, and measure the exercise-induced alteration in their redox state [119]. Quinn et al. [117] tested the intra- and inter-test reliability of FORT and FORD tests (commercially available kits) at rest, using capillary and venous whole blood and the inter-day reliability before and after sub-maximal treadmill PE. The FORT molecular kit action is based on the Fenton reaction, where hydroperoxides react with Fe^2+^ and Fe^3+^ released from the proteins, generating RO^**·**^ and ROO^**·**^, respectively. The radicals react with an amine derivative, CrNH_2_, forming a purple-coloured radical cation (CrNH_2_^+^) of which colour intensity is directly proportional to the number of hydroperoxides in the blood sample [118]. In the FORD assay, the colourless chromogen (4-amino-N,N-diethylaniline) is transformed into the stable purple-coloured chromogen radical cation in the presence of a Fe^3+^ ion. The radical cation undergoes decolourization in the presence of antioxidants in the plasma sample through reduction, losing its radical nature. The rate of the radical cation reduction is proportional to the antioxidant concentration in the blood sample.

The study of reliability, performance and utility of the FORT and FORD tests for monitoring redox perturbance induced by treadmill running and antioxidant balance by Quinn et al. [117] showed a significant positive correlation between the FORD and TAC methods (*r* = 0.53) and between F2-isoprostanes level and the FORT test (*r* = 0.48) during quiet rest and as well as between the FORD and TAC measures (*r* = 0.54) of the post-endurance exercise redox perturbance. According to the authors’ suggestion, the results showed satisfactory validation of using the FORD test as an effective method to measure non-enzymatic antioxidant status before and after exercise. Next, there is also a suggestion that both tests require future studies, which will ensure their usefulness for longitudinal monitoring of the redox homeostasis within athletes and the effect of supplementation with antioxidants. The authors emphasize that the TAC and FORD tests have limitations, because they did not measure enzymatic antioxidant activity. In addition, the methods are based on blood samples and used blood markers of OS, which are insufficient to evaluate exercise-induced stress in skeletal muscle.

Many techniques for evaluating the antioxidant activities of samples in vitro are used. However, their relevance to biological samples is often controversial, because the measurement of OS markers may be influenced by physiological redox status, type of tissue, diet, and other factors [35, 114, 120]. The methods applied for this purpose have both advantages and disadvantages concerning their sensitivity and specificity. For this reason, there is consensus in the studies that there would be a need to assess multiple markers to obtain the correct information about the effect of antioxidants on exercise-induced OS.

### Effects of antioxidants supplementation on physical exercise-induced oxidative stress in humans

Detection of perturbation in the redox balance induced by PE is crucial, especially for individuals practising sports. It allows information on a level of OS after training, an adaptation to the stress conditions, and the need for supplementation with antioxidants.

Evidence has shown that a moderately increase of ROS/RNS can cause adaptation to exercise-induced OS due to effective signal transduction [85]. In contrast, a high level of OS may cause contractile muscle disfunction, increased fatigue, extended time of recovery, and reduced sports performance [97, 106, 107, 109]. Studies have suggested that high-load resistance PE can stimulate mitochondrial biogenesis and respiratory function through myofibrillar or mitochondrial protein synthesis, depending on an individual's training status and the exercise-performed magnitude [121]. In turn, fatiguing low-load resistance PE promotes muscle hypertrophy and mitochondrial adaptation of skeletal muscle without muscle myofibrillar accretion [98]. The increased volume of mitochondria involves a smaller disturbance of the redox homeostasis in trained than in untrained individuals in response to exercise of the same intensity.

Furthermore, the muscle adaptation to endurance PE also includes a slower utilization of muscle and blood glucose, reduced lactate formation, and an increased release of free fatty acids. Thus, individuals may be more able to perform prolonged strenuous PE during endurance training [122]. Exercise performance training aims to improve the muscle's strength, speed, and agility and prevention of injury. Exercise training and antioxidants are fundamental for improving endurance capacity and sports performance [123]. To minimize OS, improve exercise performance, and prevent muscle damage by ROS/RNS, supplementation with exogenous antioxidants is common practice among endurance athletes. This strategy is derived from athletes' hope that a supplement intake may delay the onset of muscular fatigue, enhance recovery, and improve sports performance. Therefore, antioxidant supplementation has attracted attention from athletes, scientists, and supplement industries. A significant question is whether delivering antioxidant supplements, such as PCs, can improve exercise performance outcomes and what is an effect (beneficial or detrimental) of long-term supplement consumption on skeletal muscle adaptation to endurance- or resistance training [124]. There is increasing scientific evidence supporting the participation of ROS/RNS in the redox homeostasis regulation of exercise-induced blood flow, influencing exercise capacity, muscle function and performance [89]. As was shown above, ROS/RNS can play a dual role, beneficial and cell-damaging, influencing vascular function regulation. The beneficial effect of these species produced during training involves a promotion of antioxidant defense that is helpful for vascular function and adaptation to OS. Their influence depends on the tissue redox environment and individuals' redox state. However, excessive generation of ROS/RNS during intense PE disturbs the redox balance, is linked with fatigue, and decreases athletes' performance and/or a longer time recovery after exercise [92, 119].

Adapting skeletal muscle to exercise training results from increased mitochondrial volume and functional ability and is regulated by PGC-1α. Several authors have summarised the mechanism mainly based on animal studies [89, 125]. Briefly, the transcriptional response to an aerobic exercise triggers PGC1-α activation by signals from upstream kinases AMPK and p38 MAPK, followed by up-regulation of mitochondria genes and proteins. This process is considered redox-sensitive, and NOX2-induced production of mitochondrial O_2_^**·**^‾/H_2_O_2_ leads to activation of MAPK p38 [110, 125]. Furthermore, ROS/RNS production by contracting muscle also involves the cytoprotective Keap1–Nrf2 pathway and the redox-sensitive NF-κB signalling regulated by several redox-sensitive kinases. It is followed by the induction of antioxidant enzymes and catecholamines. Evidence exists that supplementation with antioxidants in exercising humans improves vascular function to maintain the redox homeostasis and help to delay muscular fatigue, and enhances endurance sport performance. On the other hand, growing findings have also shown the harmful influence of supplementing with high doses of antioxidants on skeletal muscles' response to endurance training [126].

### Vitamins C and E and N-acetylcysteine supplementation

Several studies have summarized experimental studies on the beneficial effects of commonly used dietary supplements vitamin C and E and PCs [89, 124, 127–129]. A systematic review and meta-analysis of randomized controlled trials by Clifford et al. [124] focused on the effect of vitamins C and E on exercise training adaptation. Based on 18 trials included in the analysis of aerobic exercise adaptation (*n* = 9) and resistance training adaptation (*n* = 9) (supervised exercise training through ≥ 4 weeks) found that the supplements did not reduce VO_2_max or endurance performance increase by training. They also did not affect lean mass and muscle strength following resistance training. Furthermore, a previous narrative review by Braakhuis [121] based on 12 studies found that chronic intake of vitamin C in high doses (≥ 1 g/day) impaired sports performance significantly (*n* = 4 studies) or nonsignificantly (*n* = 4 studies), and four studies found a positive effect on performance for ≤ 1-week intake of the supplement. The author concluded that vitamin C intake through diet at doses up to 250 mg/day might reduce OS and not affect training adaptation. Another review by Braakhuis and Hopkins [129] was based on 71 studies; 14 studies were devoted to supplementation with vitamin E, and nine studies investigated the effects of N-acetylcysteine (NAC) (GSH precursor) on exercise performance. These authors observed that acute vitamin E and NAC intake might increase athletes’ preformation around competition time. Still, chronic supplementation with these antioxidants may negatively affect sports performance. In addition, a current systematic review by Dervim-Lanpir et al. [125] on the effect of NAC supplementation on the hormetic response to exercise found several uncertainties in using NAC as an antioxidant supplement, emphasizing the lack of consensus on the optimal dose. It is noteworthy also to present the experimental studies focusing on reasons for the observed failure of antioxidant supplementation in reducing OS and other health benefits. For example, a placebo-controlled crossover design study by Paschalis et al. screened 100 males for vitamin C [130] and GSH [131] baseline concentrations in the blood. All examined individuals performed aerobic exercise to exhaustion and performance tests (VO_2_max, time trial, and the Wingrade test) before and after consumption of vitamin C and NAC for 30 days. The authors found that individuals with low vitamin C baseline levels had lower VO_2_max values and higher markers of OS than those with high baseline vitamin C levels; vitamin C intake increased physical performance and significantly decreased OS in the group with low baseline vitamin C levels, and nonsignificantly in individuals with high vitamin C level. Similarly, individuals with low GSH baseline levels had decreased physical performance, increased OS and impaired redox metabolism. These decreases were restored after the supplementation with NAC. In contrast, individuals with moderate and high GSH baseline levels did not experience the benefit in the context of intaking NAC supplements. These authors concluded that the efficiency of supplementation with antioxidants depends mainly on their redox status. In turn, a study by Margaritelis et al. [132] of a crossover design examined 73 individuals for plasma vitamin C and erythrocyte GSH levels and the effect of vitamin C and NAC supplements on response to OS. The authors demonstrated that treatment with 1 g/day of vitamin C or 1.2 g of NAC/day for 30 days reduced resting systemic OS levels in the groups with low vitamin C and low GSH levels compared to the groups with moderate vitamin C and GSH levels. The groups with low vitamin C and GSH levels also experienced improved performance (VO_2_max, isometric peak torque, but not the Winograd test) after vitamin C and NAC supplementation and reduced fatigue only after treatment with NAC. According to these research conclusions, OS itself is not the only reason to use supplementation with antioxidants. Furthermore, Arc-Chagnaud et al. [87] concluded that independently of the type of antioxidant supplement and form (extract, chemical content, composition), if the supplement dosing does not consider the individual’s redox status, it may not reach the benefits. Evidence has suggested that excessive intake of antioxidant supplements may reduce specific cellular signal transduction pathways induced by training which participate in chronic training adaptation to PE [133].

### Polyphenols supplementation

In recent years, several reviews and meta-analyses have quantified the effect of PC supplementation on OS and athletic performance [89, 123, 126, 128, 129]. A review of 71 studies by Braakhus and Hopkins [129], based on animal and human studies, summarized the effect of PCs, such as quercetin, resveratrol, epicatechin and beverages-containing PCs: green tea, beetroot juice with supplementation periods from acute, i.e., ranging from minutes to hours prior to a performance test to 5 months on sports performance. The authors found that quercetin exerted a small beneficial effect on performance in healthy and active rats but was potentially harmful to humans and inactive rats. Furthermore, resveratrol induced mitochondrial biogenesis and enhanced performance in the physically active rodents, but its supplementation was significantly harmful in the inactive rodents. The performance improvement versus control ranged from 128% of the maximal benefit to 81% of performance impairment (findings based on six animal studies). The authors found only one human study with resveratrol intake; findings showed a 4% statistically insignificant performance reduction in the untrained males (*n* = 27) estimated after 5 km walking and taking 250 mg/day of resveratrol for 8 weeks. Similarly, the effects of other PCs summarized in this review also showed contradicted results ranging from promising for increasing athletic performance when supplemented with cocoa epicatechin and grape extract. In contrast, the potentially harmful effects were seen in the case of green tea extract and granberry–grape seeds powder intake. The authors, referring to the optimal timing of consuming antioxidants, have concluded that much of the evidence supports an acute intake for performance benefit. Furthermore, they suggested that chronic supplementation may be followed by performance impairment except for some PCs, such as epicatechin and resveratrol, of which chronic intake in combination with training is potentially beneficial. Another review and meta-analysis by Somerville et al. [126] based on 14 studies reported increased training performance on average by 1.90% (95% CI: 0.40–3.39) among trained males supplemented with a PC dose of 688 ± 478 mg/day for at least 7 days. Moreover, a subanalysis of seven studies, where quercetin was used as a supplement showed a 2.82% (95% CI: 2.05–3.58) increase in performance. In addition, a current review by Nobari et al. [128] noticed the beneficial effects on exercise performance of drinking green tea connected with moderate intensity PE. It was seen as increased metabolism of fats and reduced energy expenditure at the same exercise intensity. It delayed fatigue in humans, owing to constituents of teas (green tea, Pu-erh tea, Pu-erh green tea). In this regard, it should be noted that a study by Nogueira et al. [134] reported that epicatechin enhances resistance against fatigue and oxidative capacity; performance improvement was larger than that caused by exercise only. A synergic effect of these two interventions on performance benefit was observed. In addition, Mafi et al.’ [135] supervised an 8-week RCT study (*n* = 62 males with sarcopenia participating in resistance training). They found that supplementation with epicatechin enhanced skeletal muscle strength and muscle mass induced by exercise training and extended training time. Evidence has shown that the effect of PCs may be depended on the type of organs. An experimental study by Yada et al. [136] found a different effect of the high dose of acacia PC supplementation on exhaustive PE-induced OS in mouse liver and skeletal muscles. The supplement administration increased OS in mouse liver regardless of exercise but decreased skeletal muscles. Furthermore, a previous study by these authors [123] reported that the PC fraction of *Tabebuia avellanedae* increased the running time until exhaustion in mice. The authors concluded that supplementing these antioxidants improved endurance capacity, regulating skeletal muscle glycogen metabolism and blood glucose levels and inhibiting exercise-induced OS. They also found that single intervention, i.e., intake of the PC fraction (without exercises), increased phosphorylation of AMPK and gene expression of deacetylase enzymes—sirtuins. The redox-related effects of a wide range of antioxidants supplementation on training adaptation were also a subject of Mason et al.’ review [89]. In this review, the authors largely focused on the commercially available supplements containing PCs, often consumed by the individuals involved in endurance training. They consider them safe even when taken in high doses. Briefly, anthocyanins exerted a mixed effect on OS and an ambiguous effect on endurance performance; astaxanthin-decreased level of OS, improved endurance performance and hindered induction of antioxidant enzymes (estimated in animals). Catechins exerted equivocal effects on OS, antioxidant enzymes level, and skeletal muscle mitochondrial biogenesis. In addition, there was no confirmation of the increased endurance performance, possible increases in fat oxidation, and decreased carbohydrate oxidation during chronic supplementation. Curcumin decreased OS in skeletal muscles and increased mitochondrial biogenesis and endurance performance (in rodents, no evidence in humans). Quercetin showed a limited effect on OS and a possible small beneficial effect on endurance exercise performance (mainly in untrained individuals). Evidence showed any effect of this supplement intake on mitochondrial biogenesis in humans. Resveratrol decreased OS in skeletal muscle and improved the level of antioxidant enzymes and exercise performance in rodents, but evidence of these outcomes in humans is limited and mixed. Referring to that group of PCs, these authors concluded that there is “insufficient supportive evidence to recommend to athletes”. The authors made the same conclusion for supplementation with vitamins A, C, E, C + E, A/β-carotene, alpha-lipoic acid, coenzyme Q10, NAC, melatonin, and microelements selenium and zinc. However, the authors have supposed that NAC dosed at < 70 mg/kg and consumed chronically over several days prior to endurance PE may benefit exercise-related outcomes. Interesting, Arc-Chagnaud et al. [87] carried out a study focusing on the role of an antioxidant/anti-inflammatory cocktail composed of several PCs, vitamin E (138 mg), selenium (80 μg) and omega-3 fatty acids (2.1 g) in the prevention of the conditioning of men skeletal muscles caused by long-term physical inactivity. The conditioning shifts the cellular redox state towards pro-oxidant reactions and OS development. For protein content quantification, samples of skeletal muscles (acquired using biopsy) were tested using the OxyBlot protein oxidation kit (detection of carbonylated proteins concentration) and immunohistochemistry. The ROS-induced muscle damage was analyzed using 4-HNE as a bioactive marker of lipid peroxidation. The authors found that the levels of carbonylated proteins were significantly decreased in the group treated with the PCs-containing cocktail compared with the placebo group. Measurements of GPx and catalase levels in muscles showed that the cocktail prevented oxidative damage without affecting the expression of the antioxidant enzymes.

Table 1 shows the representative original human studies (*n* = 15), published between January 2006 and September 2021, for the effect of phenolic products served in various forms, except for beverages containing alcohol, on exercise-formed OS, inflammation, and exercise performance.

**Table 1:** Effect of phenolic compounds supplementation on oxidative stress induced by severe exercise training in humans and exercise performance

Study, year	Participant characteristics	Polyphenols supplement	Exercise protocol	Results	Conclusions
Morillas-Ruiz et al., 2006 [40]	Sixty moderate training cyclists: PC-treated *n* = 30, placebo-treated *n* = 30 sportsmen	Beverage containing black grapes (81 g/L), raspberry (93 g/L), red currant (39 g/L) consumed before exercise	Submaximal 90 min aerobic exercise on bicycle ergometer at 70% VO_2_max	No significant changes in plasma TAS and LDL levels in both groups after exercise or supplementation. Lower increases of CK and TBARS in the supplemented group *vs* the control group. Decreased level of CO groups in PC treated group	Supplementation with PC may protect against exercise-induced OS
Sadowska-Krępa et al., 2008 [141,142]	Physical education students (*n* = 14)	Three capsules of 390 mg/day red grape skin extract (188 mg/g polyphenols plus 35 mg/g of anthocyanidins) three times/day for 6 weeks	Moderate-to-high intensity interval type swimming test (six repeats of 50 m)	Reduction of CK activity, increased GSH, uric acid, TAS in plasma, and swimming performance. Nonsignificant changes in levels of antioxidant enzymes (SOD, CAT, GSH-Px, GR)	Supplementation with red grapes skin in sport training enhanced the hemodynamic status and performance and exerted only a minor effect on activity of antioxidant defense system
Orsati et al., 2010 [139,142]	Clinical randomized double-blind placebo-controlled trial, *n* = 80 sedentary women aged 45–70 years (*n* = 40 receiving PC, *n* = 40 receiving placebo) divided into groups: Training + PC (*n* = 15); PC intake only (n = 20); training + placebo (*n* = 18); placebo intake (*n* = 18)	100 mg/day of soy isoflavone standardized extract twice per day for 9 months	Resistance dynamic exercise through 60 min, starting from lighter load at 40–50% of 1-RM, ending at 60–80% of 1RM (15 and 8–12 repetitions, respectively)	Significantly increased muscle strength (35.2%) and muscle mass (1.4%), decreased body fat in training groups. Isoflavones intake was without effect on body composition and muscle strength	Increased levels of antioxidants (daidzein, genistein) in the supplemented groups *vs* the placebo groups. A lack a synergy between resistance training and isoflavone intake in enhanced muscle strength
Skarpańska-Stejnborn et al. 2010 [138,139]	Trained male rowers (*n* = 22) aged 20.4 ± 1.1 years, PC-treated group *n* = 10, control group *n* = 12	One capsule of Panace-Vid 2000®/day (188 mg/g PC: Catechin, gallic acid, quercetin, resveratrol, 35 mg/g of anthocyanins) for 6 weeks	Repeated cycle sprint test on rowing ergometer varying, from 40 to 90% of VO_2_max	Significantly increased plasma TAC, decreased levels of GPx and lipid peroxidation products	Grapes PC enhanced the endogenous antioxidant defense
Allgrove et al., 2011 [144]	Randomized counter balanced crossover study of physically active men (*n* = 20)	Dark chocolate containing 54 mg of catechin and 44 mg of flavanols supplemented twice daily and once intake 2 h before exercise, for 2 weeks	Cycling for 90 min with varying VO_2_max from 60 to 90% for 30 s every 10 min, followed by this activity to exhaustion at 90% VO_2_max	Lowered levels of blood F2-isoprestanes at exhaustion and after 1 h of recovery, oxidized LDL before and after exercise; elevated levels of FFA in supplemented group. No significant effect of the antioxidant on IL-6, IL-10, IL-1Ra, glucose, glucagon, insulin, and cortisol levels and time to exhaustion	Supplementation with dark chocolate reduces some OS markers and increases free fatty acids mobilization after exercise
Jówko et al., 2011 [146]	A double blind, randomized placebo crossover study, male sprinters (*n* = 16) aged 21.6 ± 1.5 years PC-treated (*n* = 8), placebo-treated (*n* = 8), and vice versa	Green tea extract (980 mg of PCs) intake for 4 weeks	Repeated cycle sprint test on cycle ergometer (4 × 15 s with 1-min rest intervals), 4 weeks of strength training	Induction of increased levels of MDA, TAC and SOD in the placebo group and increases in uric acid, albumin, and CK in both groups, after exercise. Increased resting TAC level and decreased MDA and SQ levels in the supplemented group after exercise. A lack of effect on sprinters physical performance	Supplementation with green tea extract protects against OS, but does not enhance efficiency of the antioxidant enzyme systems and sprinters performance
Davison et al., 2012 [145]	Randomized counter balanced study, healthy men (*n* = 14) aged 22 ± 1 years	Dark chocolate (70% of cocoa) intake 2 h before exercise	Cycling at 53 ± 1.9 VO_2_max for approximately 2.5 h, exercise bouts (*n* = 5) separated by once week	DC increased pre-exercise plasma insulin and TAC, decreased disturbance of glucose level, and reduced slightly plasma F_2_-isoprostane level induced after prolonged exercise. There was limited effect of DC intake on immunoendocrine system, IL-6 cytokine, total leucocytes number, and neutrophil functions	Consumption of dark chocolate may influence the insulin, TAS, glucose levels and OS responses to prolonged exercise, but exerts minimal effect on immune-endocrine system
Jówko et al., 2012 [148]	Randomized double-blind placebo-controlled study of 16 soccer players aged 22.9 ± 5.5 years. PC-treated (*n* = 8), placebo-treated (*n* = 8)	640 g of green tea (500 mg of catechins, 352 mg of EGCG) or placebo administered 2 h after breakfast	Muscle-endurance test: Three sets of two strength exercises (bench, press, back, squat) performed to exhaustion with load of 60% 1-RM	Significantly increased levels of TBARS, uric acid, TAS, CK and total catechins after exercise in both groups	Supplementation with green tea PC was without effect on the OS generated by muscle-endurance test
Voduc et al., 2014 [140]	A randomized placebo-controlled double blind crossover study, 13 healthy sedentary adults (six men, seven women) aged 18–65 years	Resveratrol treatment for two 4-week periods with 2-week break: 500 mg/day in the first week and 1000 mg/day in the remaining 3 weeks	Cycling at 75% VO_2_max (four sessions) on cycle ergometer, using 20 W increments every 2 min	A small reduction in fasting glucose level, no significant changes in blood inflammatory markers and exercise capacity. High-dose resveratrol intake was followed by gastro-intestinal mild side effects	Short duration intake of resveratrol by non-obese healthy subjects was without effect on exercise-induced OS and physical performance
Jówko et al., 2015 [147]	Randomized double-blind study, 16 sprinters aged 21.6 ± 1.5 years	Green tea extract (980 mg of PCs/day) or placebo treatment for 4 weeks	Two-repeated cycle sprint tests on a cycle ergometer with submaximal load to 130–150 heart beats/min	Increased blood MDA, TAC, and SOD in the placebo group and CK activity in both tested group after exercise. Intake of PC increased TAC levels at rest and decreased SOD and MDA after exercise	Treating with PC prevents against exercise induced OS but the prevention against oxidative damage of muscle induced by exercise and an improvement in sprint performance were not noted
Toscano et al., 2015 [138]	Recreational active runners of both sex (*n* = 28) aged 39.8 ± 8.5 years, grape group *n* = 15, control group *n* = 13	Grape juice 10 mL/kg/day containing 1.82 g/L of total PC (52.28 mg/L monomeric anthocyanins) prior and after training for 28 days	Three exercise tests: a time-to-exhaustion, anaerobic threshold, and aerobic exercise	Increased blood TAC, decreased AGP and extended time to exhaustion running. Treating with PC did not exert effect on CK activity, LDL and CRP levels, and the immune system response	Supplementation with PC improves antioxidant status, may reduce inflammatory markers, and increases physical performance
Cases et al., 2017 [150]	A randomized double-blind crossover recreational active athletes (*n* = 20 men) aged 22.2 ± 2.2	Two 500 mg capsules of PerfLoad® containing 290 mg of PC and 120 mg of caffeine supplemented 60 min before exercise	The Wingate test (4 × 30-s bouts of cycling on a cycle ergometer)	Significant increases of SOD, GPx and CAT, stabilization of plasma levels of LDH and redox homeostasis. Elevated total maximal peak power output an average power in PC-treated group without much fatigue	Supplementation with PC strengthens the endogenous antioxidant status, decreases exercise-induced stress, and improves physical performance
Giolo et al., 2018 [143]	Double-blinded randomized placebo-controlled trial, 32 healthy non-obese postmenopausal women aged 50–70 years, exercising + placebo (*n* = 15), exercising + PC treatment (*n* = 17)	100 mg of isoflavones/day from soya bean (daidzein, 93.5 mg: genistein, 3.3 mg; glycitein, 3.2 mg) for 10 weeks	Combined aerobic exercise (20 min) and resistance exercise (20 min) during one session (50 min) on a treadmill at 85% HRmax for 10 weeks (30 sessions)	No significant difference between the both groups in levels of the blood inflammatory cytokines (IL-1β, IL-6, IL-8, IL-10, IL-12p70), TNF-α, OS markers (TAC, SOD, TBARS). Exercise induced increased IL-8 levels correlated with reduction of total cholesterol in both groups	PC intake after exercise showed no anti-inflammatory and OS reductive actions and was without effect on plasma lipid profile. Isoflavone treatment had no additional beneficial effect on exercises-mediated responses
de Lima Tavares Toscano et al., 2020 [137]	A randomized, crossover, double-blind study of healthy male runners (*n* = 14) aged 39 ± 9 years involved in the experimental and control group	3106.6 mg/L PC from grape containing flavanols, flavonols, phenolic acid, and stilbenes. A single dose of 10 mL/kg/day before the run (for 28 days)	Two running tests to exhaustion (80% VO_2_max, 3.2 km). VO_2_max = 55.9 ± 6.5 mL/kg/min for 68.4 ± 29.7 min in the supplemented group and 59.2 ± 27.2 min in the placebo group	Significantly increased TAC (43.6%) after exercise in the supplemented group *vs* the basal level. No significant changes in levels of the blood inflammatory markers (MDA, CK, hs-CRP, AGP, LDH) in both tested groups	Grape juice ingestion increased antioxidant status and physical performance by increasing run time to exhaustion
Zhang et al., 2020 [149]	Double-blinded randomized controlled study. 11 male and 13 female non-obese subjects	20.6 mg/day avenanthramides from oat cookies consumption (206 mg/kg) for 8 weeks	Downhill running on treadmill at 75% of HRmax (four bouts of running with 15 min each at -10% grade separated by 5-min rest)	Significantly reduced the blood levels of CK, neutrophil respiratory burst, granulocyte colony-stimulating factor, IL-6 and IL-1Ra rest	PC ingestion reduced levels of inflammatory cytokines and chemokines, decreased expression of cell adhesion molecules, and protected against muscular exertion

*PC* phenolic compounds, *TAS* total antioxidant status, *TAC* total antioxidant capacity, *SOD* superoxide dismutase, *CAT* catalases, *OS* oxidative stress, *CK* creatinine kinase, *MDA* malonaldehyde, *SQ* semiquinone, *TBARS* thiobarbituric acid reactive substances, *VO*_*2*_*max* maximal oxygen intake, *IL-6* interleukin-6, *IL-8* interleukin-8, *IL-10* interleukin-10, *IL-12p70* interleukin-12 antibody, *IL-1Ra* interleukin-1-receptor antagonist, *IL-1β* interleukin-1β, *TNF-α* tumor necrosis factor alpha, *hs-CRP* high sensitivity C reactive protein, *AGP* alpha 1-acid glycoprotein, *LDH* lactate dehydrogenase, *GPx* glutathione peroxidase, *GR* glutathione reductase, GSH glutathione, *LDL* low density lipoproteins, *HDL* high density lipoproteins, *FFA* free fatty acids, 1*RM* one repetition maximum, *HR* maximum heart rate, *EGCG* epigallacatechin-3-gallate, *CO* carbonyl group, *DC* dark chocolade

The studies differ from one another in terms of the PCs source: grapes [40, 137,141] ; soy extract [142, 143] ; cocoa [144, 145] ; leaf tea [146,148] ; oat [149]; and forms of their intake (capsule of PCs rich extracts Panace-Vid2000^®^ [139] or PerfLoad^®^ [150] juice, beverage, chocolate, tea); a supplement dose and timing; exercise type, intensity, duration, as well as training status. Most of the individuals in this study were physically active. They participated in various types of endurance sports training, such as cycle ergometer [40, 139, 140, 144,147], sprint exercise [137, 138], muscle endurance strength exercise [142, 148] , treadmill [143, 149] and swimming [141] . The exercisers were mainly engaged in an exercise of the maximal oxygen uptake (VO_2_max) or VO_2_ submaximal. Different markers of OS and inflammation, including pro- and anti-inflammatory cytokines and enzymatic antioxidants, were detected using the blood samples in 14 of 15 presented studies. Twelve [40, 137, 138, 141, 142, 144–150] of 15 presented experimental studies observed reduction of OS markers or enhancements of TAC and TAS and reduction of inflammatory factors. Eight [137, 138, 140–142, 146, 147, 150] of 15 studies tested the effect of PCs supplementation on physical performance, observing a lack of significant effect in three studies [140, 146, 147], four studies [137, 138, 141, 150] found improvement of physical performance compared with control groups. One study [142] reported a significant increase in muscle strength caused only by training. In turn, a study by Zhang et al. [149] reported the PC supplement protection against exercise-induced muscle damage, which could have had a negative impact on endurance running performance.

Interestingly, some studies presented in Table 1 observed significant decreases only in a few OS markers and unchanged other markers [137, 144, 145]. For example, a study by Allgrove et al. [144] reported a decrease in F_2_-isoprostane and LDL levels but a lack of the significant effect of PC supplementation on glucose, glucagon, insulin levels, inflammatory cytokines, and interleukin-1 receptor antagonist (IL-1Ra) gene. Furthermore, a study by Davison et al. [145] observed also reduced F2-isoprostane and the decreased glucose level induced by exercise but the limited effect of supplementation on an immunoendocrine system, IL-6, leukocyte, and neutrophil activities. Next, de Lima Tavares-Toscano et al. [137] observed strongly increased TAC and exercise performance, but the markers of inflammation were unchanged in the supplemented group compared with the control group. Interesting, supplementation with a mixture of isolated isoflavones: daidzein, genistein, and glycitein in a study by Giolo et al. [143] and with resveratrol alone in a study by Voduc et al. [140] caused in exercisers insignificant changes in the plasma OS as well in the inflammatory markers after supplementation with these PCs. It should be underlined that the TAC technique only indicates the summarized action of low-molecular-weight antioxidants present due to a diet rich in fruits and vegetables and/or supplementation. In turn, Voduc et al. [140] observed that intake of high doses of resveratrol resulted in side effects (small increases in total cholesterol, triglycerides, and liver enzymes).

On the contrary, treatment with low doses of this PC produced a noticeable reduction in fasting glucose, as was reported by Bowtel and Kelly [66]. PCs as high-molecular-weight compounds present in plasma at low concentrations cannot compete with the direct ROS/RNS scavengers having low-molecular-weight, such as vitamins C and E, ascorbate or tocopherols present at high concentrations. Moreover, all the tested studies were acute exercise studies probably accompanied by high levels of ROS production, especially in contracting muscles. Thus the levels of the OS and inflammation markers might be even higher than before exercise. The duration of the supplementation protocols ranged from acute intake to 10 weeks. There is not possible from these findings to suggest how long-term PC intake would have affected chronic training adaptation. It is caused by the different experimental conditions, such as substantial variability in the type, daily dose, form, and producers of these supplements across the studies and training protocols. In addition, different analytical methods were applied to identify the exercise-mediated disturbance of the oxidative redox status markers. There is also diverse literature finding in this area. Evidence has shown that acute PC intake resulted in exercise performance benefits in humans, whereas chronic supplement intake might cause performance impairment. However, data from animal studies have presented enhanced training performance in chronically supplemented animals with epicatechin or resveratrol [129]. The findings listed in Table 1 also show that physical training protocols varied significantly between studies. In addition, the tested subjects differed regarding their redox state at rest. As mentioned above, Paschalis and co-workers’ experimental studies [130, 131] of individuals with low, moderate, and high resting levels of blood GSH and vitamin C levels, engaged in PE and treated with NAC or vitamin C showed that the individuals had experienced a reduction of OS and enhancement of physical performance only in the groups with low resting GSH and vitamin C concentrations. It suggests that the beneficial effect of antioxidant supplementation in the individuals with a higher level of antioxidants in the rest, i.e., having already the optimal redox balance, may not be observed when measuring OS markers, e.g., as it was seen in the studies by Jówko et al. [148] and Voduc et al. [140]. Notably, the concentration of the antioxidants in the body depends on the type of tissue [151, 152]. Tissue delivery of PCs has rarely been measured compared with the number of studies that reported the levels of these compounds in plasma. We know only two studies examined PC levels as a supplement in skeletal muscles; both used animal tissues [151, 152]. A study by Andres-Lacueva [151] found the highest concentration of resveratrol and its metabolites in the liver, followed by detectable amounts in adipose tissue and below the limit of detection in skeletal muscles in supplemented rats with 6,30 or 60 mg/kg body weight/day of resveratrol for 6 weeks. This finding confirmed data from a previous study by Azorin-Ortuño et al. [152] that analyzed tissue distribution of resveratrol and its metabolites in pigs after 6 h of intragastric administration (59 mg/kg body weight/day for 6 weeks). Findings showed that approximately 74.5% of the total resveratrol administrated was detected as resveratrol and dihydroresveratrol metabolites, of which 65.1% along the gastrointestinal tract, followed by 7.7% in urine, 1.2% in bile, and only 0.5% in the organs: ovaries, uterus, pancreas, muscle, and others. These findings demonstrate a very low level of PC concentration in skeletal muscles, which should be considered during supplementation.

As mentioned above, the skeletal muscles are the primary generators of ROS during their daily PA and during excessive PE or the ageing process [96, 97]. In addition, a concentration of ROS is strongly elevated in muscles after muscle injury and regeneration [153]. Therefore, the studies addressing this issue should focus on a safe dose of PC supplementation and evaluation of OS within skeletal muscles, as the evaluation of the OS and inflammation markers in the blood is an insufficient substitute for determination processes occurring within muscles. A proper estimation of the role of PC supplementation in preventing OS and improving sports performance should put the proper measure of an individual’s redox status at rest to avoid the excessive dose of the supplement intake. A lack of estimation of the rest redox balance may explain partially the inconsistencies between findings on inhibition of exercise-induced OS by the administration of the antioxidant in several studies (reviewed by Kawamura and Muraoko [97]). Measurements of the level of antioxidants in skeletal muscles at the cellular level before and after PC supplementation are recommended when the level of exercise-induced OS is evaluated.

### Methodological limitations of the existing evidence and future research trends

The existing literature in this area is flawed by several limitations that may impact the current evidence. First, a poorly PC characterisation was found in some studies. The authors studied the supplements, which were often characterised using non-specific analytical techniques for detecting and characterising the total phenolic content and the profiling of PCs when isolated from plant species or even when the supplement characterisation was reported. It might limit the accuracy of the reported optimal doses and blend, having potency to introduce bias, especially since the antioxidant power and content of phenolic compounds in plants depend on several factors (e.g., geographical region, climate, storing conditions). However, bias is inherent in the majority of systematic reviews. Therefore, special attention should be given to the standardisation of PC content in the supplement that requires specific analytical methods, such as high-performance liquid chromatography, mass spectrometry and UV spectrometry [154]. For the critical analysis of the supplementation protocols, a study by Sabou et al. is illustrative [154].

Furthermore, even if the analytical characterisation of the PC supplement was done correctly, a high variation in the supplementation protocols was seen across conducted studies. Another influencing factor is athletes’ diet. Given the prevalence of polyphenolic substances in the human diet, determining the nutritional profiles of athletes presiding training is required. Due to the high variability of the PC component in food, individuals might under- or over-reporting intake of the food antioxidants. Thus the accurate quantification of nutrients based on the standard and validation methods should be used. Knowledge of the limited bioavailability, the short half-life period, and a broad spectrum of PC biochemical effects [40] is essential when choosing supplement intake timing. The current research does not indicate recommendations concerning PC supplementation timing and daytime.

Observational studies of PE and OS also suffer from limitations in measuring physical effort due to the complexity of this relationship. A variety of physical training protocols and assessment methods across studies are applied in the reviewed studies. The methods used to determine PE and training protocols also varied between studies. They could not be sufficiently reliable and valid to assess the effect of endurance training on desired outcomes. Type of PE, intensity, volume, type of muscle contraction and several muscle groups involved in exercise and duration of exercise protocols are valuable factors that might influence the reported findings when considering the effect of PC intake on desired outcomes [65]. These are factors that should also be analysed in future research. The observational studies analysed in this review showed a large diversity of analytical assays applied for determining OS and inflammation markers as well as TAC and TAS evaluation. In addition, all studies shown in Table 1 analysed only the blood markers to evaluate OS and inflammation that are insufficient to determine the level of OS in skeletal muscles. It is an important practical limitation and a technical challenge for the applied sports sciences to develop less invasive methods for muscle biopsies. Other challenges deal with universal methods for detecting ROS levels to measure alterations in the redox homeostasis induced by severe training and the effectiveness of the antioxidant supplementation.

Moreover, the included studies were conducted mainly on small sample sizes (≤ 17 athletes) with two exceptions [40, 142] and were mostly performed in men, although also limiting other literature reviews in this area. Only a little research has been focused on women on the whole [155]. Females may have a higher level of endogenous antioxidants than males due to the superior anti-inflammatory estrogens level as well as the role of mitochondrial estrogen receptors in the preservation and regulation of mitochondrial function [156]. Due to these limitations, among other unidentified, recommendation of the PC blend, optimal dose, and timing is currently impossible.

## Conclusions

This article demonstrates the chemical structure—antioxidant relationship and molecular mechanisms responsible for antioxidant and anti-inflammatory activities of PCs, as well as the representative experimental studies on their effect on severe exercise-induced OS in humans and exercise performance. In addition, an overview of the biological properties of PCs, a disturbance in the antioxidant–prooxidant balance induced by acute high-intensity PE followed by OS, and measurement of TAS, TAC, and markers of biomolecules damage are discussed. Evidence suggests that severe PE can lead to muscle inflammation and tissue damage, positively correlated with the release of the proinflammatory cytokines and the increased CRP in athletes; these processes are confirmed in our study. The key factors influencing the OS are exercise intensity, duration, exercise training, organism adaptation, a diet concerning antioxidant content, and the level of an individual’s redox state at rest. PCs are well recognised as highly effective scavengers of free radicals and other non-radical ROS/RNS in vitro. Evidence from animal research and a limited number of studies in humans performed in a double-blind method shows that due to the poor bioavailability of PCs, and their low concentration in peripheral tissues, especially in the skeletal muscles, thus they cannot compete with low molecular-weight endogenous antioxidants as inhibitors of ROS/RNS. However, their physiological ability to induce endogenous antioxidant gene expression and regulate ROS production by enzymes and redox-related transcription factors cause PCs to be the essential reducers of OS and inflammation and protect lipids, proteins, and nucleic acid damage. The presented experimental studies have found the utility of PC intake in maintaining cellular homeostasis in individuals engaged in acute high-intensity PE, showing enhancement of TAS and TAC, increased concentration of a few antioxidant enzymes and the GSH/GSSG ratio. The literature findings are limited concerning the supplement type, effective and safe dose, and intake duration. In addition, limited studies have examined PC intake on long-term training-induced adaptations to PE. Evidence shows that the relationship between PE, OS and PC supplementation is very complex and influenced by many factors. Therefore, the exact mechanisms linked with exercise-related disturbance in the cellular redox homeostasis and the beneficial role of phenolic antioxidants in this process are not yet fully understood.

It is now evident that PCs have high antioxidant potential and an ability to improve the physiological and physical functions of athletes’ skeletal muscles. However, evidence on the relationship between PC supplementation during endurance training with exercise performance provides controversial findings. Therefore, further observational and clinical studies with a larger sample size of a double-blind design and placebo control group needed to be continued to provide a type of polyphenol supplement, its optimal safe dose and timing of supplementation. A proper analysis of total PC content and dietary assessment, standardised protocols of PC supplementation and controlled physical training programs for different sports disciplines, considering the individual’s redox state at rest and a proper biochemical analysis of OS and inflammation markers in skeletal muscles should be addressed. Additional research is needed to continue work on advances in novel noninvasive and more specific techniques to measure OS markers and levels of tissue antioxidants in skeletal muscle in response to exercise-induced ROS production and performance outcomes.

The summarised in our review, current knowledge in the chemistry and biochemistry of PCs may help to coach staff and athletes to incorporate safe and effective protocols of supplementation and exercise training. It would be helpful in a better understanding of the mechanisms of the effect of PCs on acute exercise-induced training performance.

## Data Availability

Not applicable.

## Abbreviations

^1^O_2_: Singlet oxygen
1RM: One repetition maximum
8-OHdG: 8-Hydroxydeoxyguanosine
AGP: Alpha 1-acid glycoprotein
AMPK: AMP-activated protein kinase
AP-1: Factor activator protein
CAT: Catalases
CK: Creatinine kinase
COX1/2: Cyclo-oxygenase enzymes 1 and 2
CRP: C-reactive protein
EGCG: Epigallacatechin-3-gallate
ELISA: Enzyme-linked immunosorbent assay kits
ESR: Electron spin resonance spectroscopy
FFA: Free fatty acids
FORD: Free oxygen radical defence
FORT: Free oxygen radical test
GPx: Glutathione peroxidase
GR: Glutathione reductase
GSH: Glutathione
GSSG: Glutathione disulphide
H_2_O_2_: Hydrogen peroxide
HDL: High density lipoproteins
HO: Hydroxyl radical
HOCl: Hypochlorous acid
HR: Maximum heart rate
hs-CRP: High sensitivity C reactive protein
IL-12p70: Interleukin-12 antibody
IL-1Ra: Interleukin-1-receptor antagonist
IL: Interleukin;
LDH: Lactate dehydrogenase;
LDL: Low density lipoproteins
LOX: 5-Lipoxygenase;
MAPK: Mitogen-activated protein kinase;
MDA: Malonaldehyde
Me^n^: Metal ion
NAC: N-acetylcysteine
NADPH: Nicotinamide adenine dinucleotide phosphate
NF-κB: Nuclear factor kappa B
NK: Natural killer
NO: Nitrogen monoxide
Nox2: NADPH oxidase 2
Nox4: NADPH oxidase 4
Nrf2: Nuclear factor erythroid-2related factor 2
O2 ®: Superoxide anion radical
OCl‾: Hypochlorite ion
PGC1-α: Peroxisome proliferator-activated receptor gamma co-activator 1-α
ONOO ®: Peroxynitrite
OS: Oxidative stress
P13K: Phosphatidylinositol 3-kinase
PA: Physical activity
PC: Polyphenol compound
PCs: Polyphenols
PGC-1α: Receptor gamma coactivator 1-alpha
PGE-2: Prostaglandin E2
PLA_2_: Phospholipases A2
R: Free radical
RCT: Randomized controlled trial
RNS: Reactive nitrogen species
ROO: Peroxyl radical
ROS: Reactive oxygen species
SOD: Superoxide dismutase
SQ: Semiquinone
TAC: Total antioxidant capacity
TAS: Total antioxidant status
TBARS: Thiobarbituric acid reactive substances
TGF-β: Transforming growth factor beta
TNF-α: Tumor necrosis factor alpha
VO_2_max: Maximal oxygen intake
